# Pre-clinical pharmacology of AZD3965, a selective inhibitor of MCT1: DLBCL, NHL and Burkitt’s lymphoma anti-tumor activity

**DOI:** 10.18632/oncotarget.18215

**Published:** 2017-05-25

**Authors:** Nicola J. Curtis, Lorraine Mooney, Lorna Hopcroft, Filippos Michopoulos, Nichola Whalley, Haihong Zhong, Clare Murray, Armelle Logie, Mitchell Revill, Kate F. Byth, Amanda D. Benjamin, Mike A. Firth, Stephen Green, Paul D. Smith, Susan E. Critchlow

**Affiliations:** ^1^ iMED Oncology, AstraZeneca, Alderley Park, Cheshire, UK; ^2^ iMED Oncology, AstraZeneca, Cambridge, UK; ^3^ MedImmune, One MedImmune Way, Gaithersburg, MD, USA; ^4^ iMED DSM, AstraZeneca, Cambridge, UK; ^5^ iMED Oncology, AstraZeneca, Gatehouse Park, Waltham, Massachusetts, MA, USA; ^6^ C4X Discovery, Manchester, UK

**Keywords:** AZD3965, MCT1, metabolism, lactate

## Abstract

Tumors frequently display a glycolytic phenotype with increased flux through glycolysis and concomitant synthesis of lactate. To maintain glycolytic flux and prevent intracellular acidification, tumors efflux lactate via lactate transporters (MCT1-4). Inhibitors of lactate transport have the potential to inhibit glycolysis and tumor growth. We developed a small molecule inhibitor of MCT1 (AZD3965) and assessed its activity across a panel of cell lines. We explored its antitumor activity as monotherapy and in combination with doxorubicin or rituximab. AZD3965 is a potent inhibitor of MCT1 with activity against MCT2 but selectivity over MCT3 and MCT4. *In vitro*, AZD3965 inhibited the growth of a range of cell lines especially haematological cells. Inhibition of MCT1 by AZD3965 inhibited lactate efflux and resulted in accumulation of glycolytic intermediates. *In vivo*, AZD3965 caused lactate accumulation in the Raji Burkitt’s lymphoma model and significant tumor growth inhibition. Moreover, AZD3965 can be combined with doxorubicin or rituximab, components of the R-CHOP standard-of-care in DLBCL and Burkitt’s lymphoma. Finally, combining lactate transport inhibition by AZD3965 with GLS1 inhibition *in vitro*, enhanced cell growth inhibition and cell death compared to monotherapy treatment. The ability to combine AZD3965 with novel, and standard-of-care inhibitors offers novel combination opportunities in haematological cancers.

## INTRODUCTION

Cancer cells frequently rely on glycolysis, rather than oxidative phosphorylation, for ATP generation, a phenomenon termed the Warburg effect [1]. Although increased glycolytic flux drives the production of ATP and intermediates necessary for anabolic pathways [2], a direct consequence is concomitantly increasing intracellular lactate levels, a metabolic dead end. To avoid intracellular acidification and feedback inhibition of glycolysis, intracellular lactate homeostasis in both normal and cancer cells is maintained by members of the monocarboxylate transporter (MCT) family: MCT1 (solute carrier family 16 (monocarboxylate transporter), member 1) *(SLC16A1)*, MCT2 *(SLC16A7)*, MCT3 *(SLC16A8)* and MCT4 *(SLC16A3)* [3]. MCT1, MCT2, MCT3 and MCT4 are expressed in the plasma membrane and are predicted to have 12 transmembrane helices. Functional expression of the lactate transporters require the single transmembrane ancillary proteins embigin (also known as GP70) or basigin (CD147, OX-47 or EMMPRIN) [4]. Kinetic properties of the transporters determines the role of each family in lactate transport - MCT1 can facilitate both lactate import and export, whilst MCT4 exports lactate [3]. High MCT1 expression has been reported across a range of solid tumors [5], including bladder carcinoma [6], gastrointestinal stromal tumors [7] and clear cell renal carcinoma [8]. *In vitro,* MCT1 silencing has been shown to suppress cell growth and induce apoptosis in malignant pleural mesothelioma [9], colon carcinoma [10] and glioma [11] cell lines. MCT1 has also been demonstrated to play a key role in the phenomena of lactate stromal shuttling where by lactate produced by tumor cells can be taken up by surrounding stromal and oxygenated tumor cells resulting in regeneration of pyruvate to fuel oxidative phosphorylation [12, 13].

A number of studies have demonstrated that c-MYC regulates MCT1 expression. Activation of c-MYC increases expression of MCT1 in human fibroblasts [14] and over-expression of c-MYC also increases expression of MCT1 in human breast epithelial cells [15]. Data in the Eμ-MYC transgenic mouse model of human B lymphoma confirms that MYC (V-MYC avian myelocytomatosis viral oncogene homolog) regulates MCT1 expression [16]. A canonical MYC- binding site has been identified within the MCT1 promoter region, and two non-canonical sites in tandem within the first intron of MCT2 [17]. Chromatin immunoprecipitation (ChIP) assays confirm that both c-MYC and N-MYC are recruited to the MCT1 promoter and the first intron of the MCT2 gene. Furthermore, MYC transcriptionally represses miR29A and miR29c, resulting in enhanced expression of MCT1. MYC rearrangements have been identified in 14% of newly diagnosed Diffuse large B cell lymphoma (DLBCL) and represent a subset of DLBCL with aggressive behavior and poor overall outcomes when treated with first line therapy [18, 19]. First line DLBCL therapy consisting of a combination of chemotherapy with cyclophosphamide, doxorubicin, vincristine and prednisolone (CHOP) was established almost 40 years ago [20]. R-CHOP; rituximab (an anti-CD-20 monoclonal antibody) combined with CHOP, improved progression free survival (PFS) and overall survival (OS) by 10-15% compared with CHOP alone and is currently first line therapy for DLBCL [21-23]. However, although highly effective for young patients, 40-50% of patients relapse after front-line therapy and therapy failure remains a significant challenge for DLBCL. 5-15% of DLBCL cases have been reported to carry MYC translocations [24] with concomitant high levels of MCT1 and MYC mRNA [16], highlighting that inhibition of MCT1 may represent a novel strategy to target MYC-dependent DLBCL.

Several MCT1 inhibitors have been reported including α-cyano-4-hydroxycinnamate (CHC) analogs [25] and stilbene disulfonates such as 4,4’-di-isothiocyanostilbene-2,2’-disulfonate (DIDS) and 4,4’-dibenzamidostilbene-2,2’-disulfonate (DBDS) [26] but are not selective inhibitors. Specifically, CHC is a more potent inhibitor of the mitochondrial pyruvate carrier (MPC) than of MCT1 [27] and DBDS a more potent inhibitor of the chloride/bicarbonate exchanger AE1 [28]. AR-C155858, representative of a new class of specific and extremely high-affinity inhibitors of MCT1, demonstrates a Ki of 2 nM against MCT1 in rat erythrocytes [29-31]. These compounds were originally identified as potent inhibitors of T-lymphocyte proliferation and were subsequently shown to bind to MCT1 and MCT2, but not MCT4. T lymphocyte activation and proliferation is accompanied by a significant (up to 14-fold) increase in glycolytic rate and lactate production. Inhibition of lactate efflux by AR-C155858 suppresses T lymphocyte function [30]. More recently, the anti-tumour drug lonidamine (LND), has been demonstrated to cooperatively inhibit L-lactate tranport by MCT1, MCT2 and MCT4 but with low potency (K_0.5_ of 36-40 µM) [32]. Similar to CHC, lonidamine displays potent inhibition of the mitochondrial pyruvate carrier, MPC [32].

**Figure 1:**
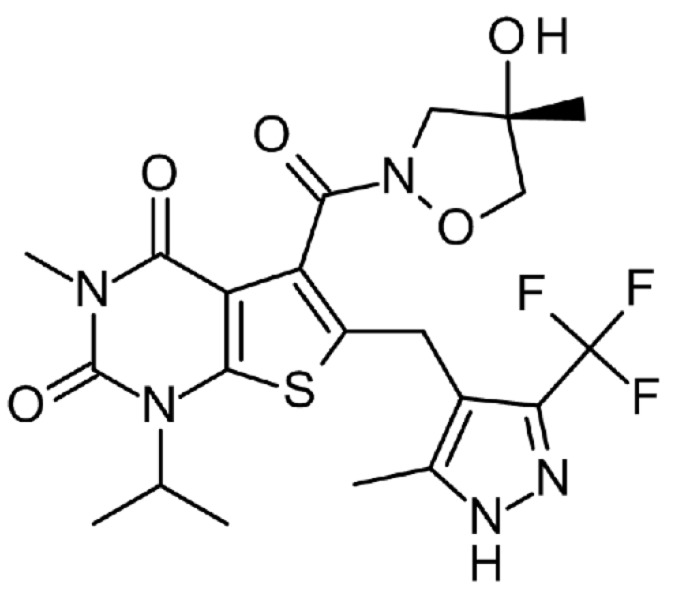
AZD3965 chemical structure

Here, we describe the pharmacological properties of AZD3965 a close analogue of AR-C155858 (Figure 1), a potent inhibitor of MCT1. We demonstrate the anti-tumor activity of AZD3965 *in vitro* in DLBCL, Non Hodgkin’s lymphoma (NHL) and Burkitt’s lymphoma cell lines and *in vivo* in the Raji xenograft model. We demonstrate enhanced *in vitro* DLBCL apoptosis following the sequential combination of AZD3965 with doxorubicin and significant inhibition of Raji xenograft growth following the concurrent administration of AZD3965 and doxorubicin. Furthermore rituximab, another integral component of the R-CHOP regimen, promotes tumour stasis in combination with AZD3965 in the Raji xenograft model. In addition, we propose a new potential therapeutic opportunity of targeting the high metabolic dependency of DLBCL, through combining AZD3965 with inhibitors of Glutaminase (GLS).

## RESULTS

### AZD3965 demonstrates high affinity and selectivity to MCT1

**Figure 2:**
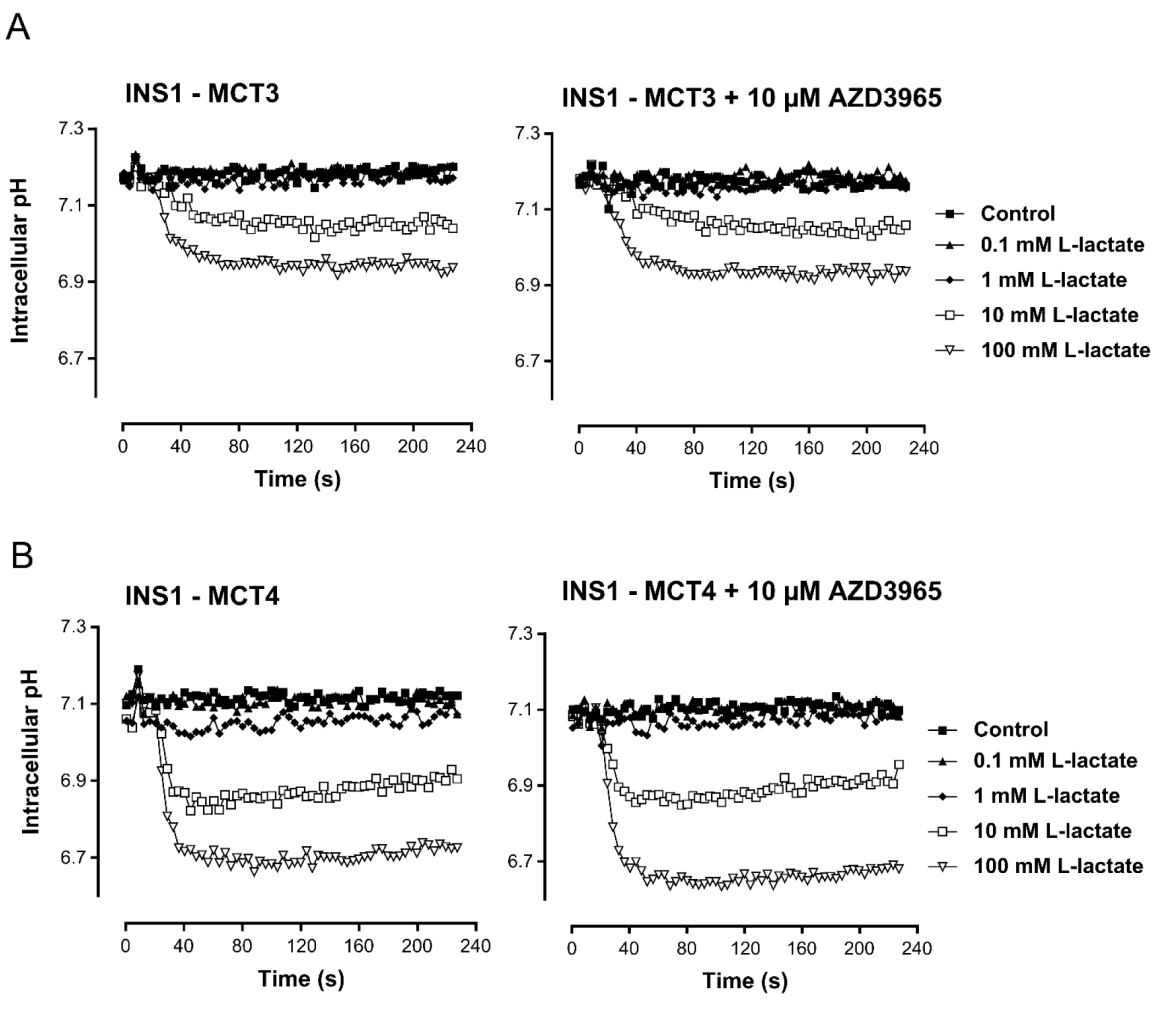
AZD3965 inhibition of lactate transport is not mediated through MCT3 or MCT4 Inhibition of lactate uptake by AZD3965 in rat pancreatic INS1 cells expressing human **A.** MCT3 or **B.** MCT4. Lactate transport mediated by MCT3 and MCT4 was not inhibited by pre-incubation of the cells for 1 hr with 10 μM AZD3965.

AZD3965 bound to human MCT1 with high affinity and potency (pKi 8.8) (Table 1A). Furthermore, AZD3965 demonstrated a selectivity ratio of approximately 6- fold against MCT2 (Table 1B). In order to confirm the selectivity of AZD3965, human MCT3 or MCT4 was expressed in the MCT1, MCT2, MCT3 and MCT4 null rat pancreatic INS1 cell line. Lactate transport into these cells was measured using the pH- sensitive fluorescent dye BCECF ((2’,7’-Bis-(2-Carboxyethyl)-5-(and-6)-Carboxyfluorescein, Acetoxymethyl Ester)) to detect rapid decreases in intracellular pH following proton-linked lactate transport (Figure 2). Previous studies demonstrated that the addition of exogenous L-lactate caused no change in pHi in control cells expressing the pcDNA3 vector [30]. Lactate transport mediated by MCT3 or MCT4 was not inhibited by pre-incubation of the cells for 1 hr with 10 μM AZD3965. Taken together, these data demonstrate that AZD3965 is a potent inhibitor of MCT1 with additional activity against MCT2.

Table 1AZD3965 MCT1 and MCT2 binding affinity.

**A:** 

	AZD3965
MCT1 Binding affinity pKi (+/- s.e.)	8.80 (0.13) (*n*=6)
MCT1 Binding affinity Ki (nM)	1.6 (*n*=6)

**B:** 

	AZD3965
MCT1 Binding affinity Ki (nM)	3.2 (*n*=2)
MCT2 Binding affinity Ki (nM)	20.0 (*n*=2)

**A.** AZD3965 binding affinity to wheatgerm agglutinin SPA beads coated with Jurkat cell membranes and incubated with [^125^I]-MCT1 ligand. B. AZD3965 binding affinity to yeast membranes expressing recombinant human MCT1 or human MCT2 by displacement of [^3^H]-MCT1 or MCT2 ligand.

### AZD3965 inhibits growth of multiple lymphoma cell lines *in vitro*

**Figure 3:**
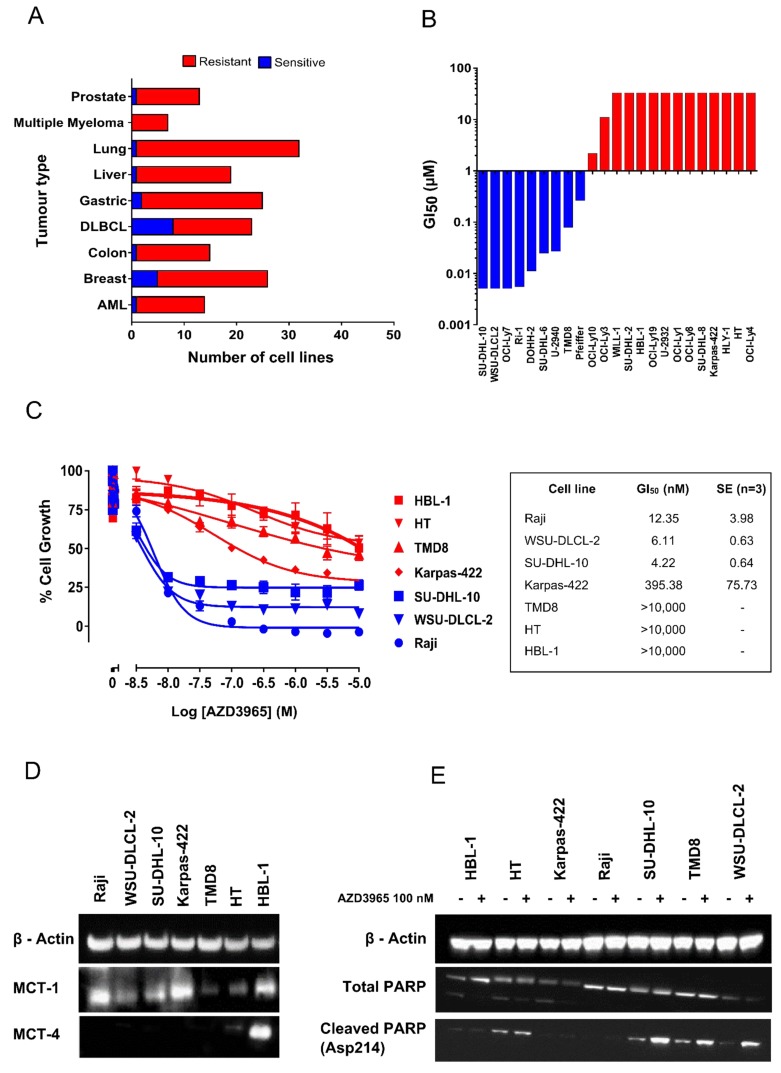
AZD3965 inhibits DLBCL, NHL and Burkitt’s lymphoma cell growth as assessed through MTS and cell number **A.** AZD3965 monotherapy activity across solid and haematological tumor cell lines. Cell growth was assessed through MTS cell viability 72 hr post AZD3965 treatment. Sensitivity was defined as GI_50_ <100 nM. (*n* = 3 independent experiments). **B.** AZD3965 DLBCL monotherapy phenotypic activity. Cell growth was assessed through MTS cell viability 72 hr post AZD3965 treatment. GI_50_ (*n* = 3 independent experiments) presented as a GI_50_ water fall plot. **C.** AZD3965 monotherapy phenotypic activity. Cell growth was assessed through viable cell number 72 hr post AZD3965 treatment. GI_50_ values +/- s.e. **D.** Western blot analysis of whole cell lysate MCT1 and MCT4. E). Western blot analysis of whole cell lysate cleaved PARP following 48 hr 100 nM AZD3965. Data representative of 3 independent experiments.

The anti-proliferative activity of AZD3965 was assessed by measuring the impact on growth on a panel of 174 cell lines derived from solid and haematologic tumors, using a standard MTS proliferation endpoint*.* Cell lines with a GI_50_ <100 nM were classified as sensitive, whereas those with a GI_50_ > 100 nM were classified as resistant (Figure 3A). Few solid tumor cell lines demonstrated monotherapy activity. However, the highest frequency of sensitivity was seen in a range of DLBCL cell lines. Moreover, detailed profiling across a further panel of DLBCL and NHL cell lines by a standard MTS growth assay confirmed that a significant number of cell lines demonstrated exquisite monotherapy sensitivity to AZD3965 with GI_50_ <100 nM (Figure 3B). MTS assay measures the bio-reduction of a tetrazolium compound through intracellular NADPH or NADH produced by dehydrogenase enzymes in metabolically active cells and therefore may not be a robust readout of cell number. To further confirm the phenotypic efficacy of AZD3965 and to build understanding of mechanisms that determine sensitivity to AZD3965, a panel of DLBCL, NHL and Burkitt’s lymphoma cell lines (Supplementary Table 1) positive for MCT1 protein expression were assayed for cell number following 72 hr incubation with AZD3965. The Raji, SU-DHL-10 and WSU-DLCL-2 cell lines were confirmed as demonstrating exquisite sensitivity to AZD3965 monotherapy with GI_50_ values <100 nM (Figure 3C). Near complete cytostasis with > 100 nM AZD3965 was identified in the Raji cell line. The Raji, SU-DHL-10 and WSU-DLCL-2 cell lines all demonstrate high MCT1 protein expression in the absence of MCT4 (Figure 3D). Karpas-422 is relatively phenotypically sensitive to AZD3965 with the TMD8, HT and HBL-1 cell lines all demonstrating relative phenotypic resistance to AZD3965. Drug-induced cell death was confirmed in the WSU-DLCL-2, SU-DHL-10 and TMD8 cell lines by exposing the cells for 48 hr to 100 nM AZD3965 and monitoring cleaved PARP (Figure 3E). TMD8 has the lowest level of MCT1 expression in the panel, whilst both the HT and HBL-1 cell lines are positive for both MCT1 and MCT4 expression suggesting that MCT4 may compensate for lactate transport in the HT and HBL-1 cell lines [33]. Taken together, these data confirm that AZD3965 inhibits the growth of a range of lymphoma cell lines and suggest that MCT1 and MCT4 expression status influences inhibitor sensitivity.

### AZD3965 inhibits lactate transport

**Figure 4:**
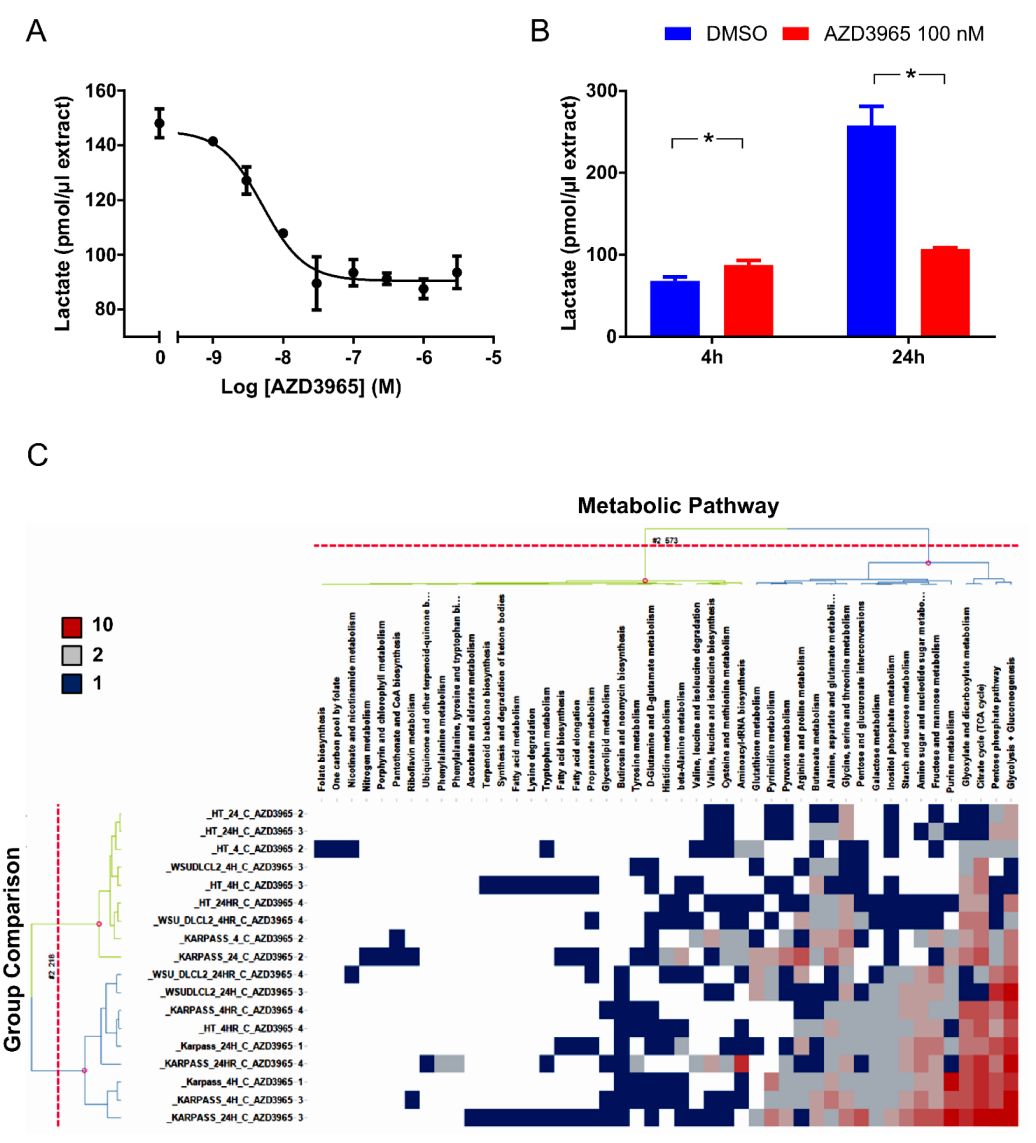

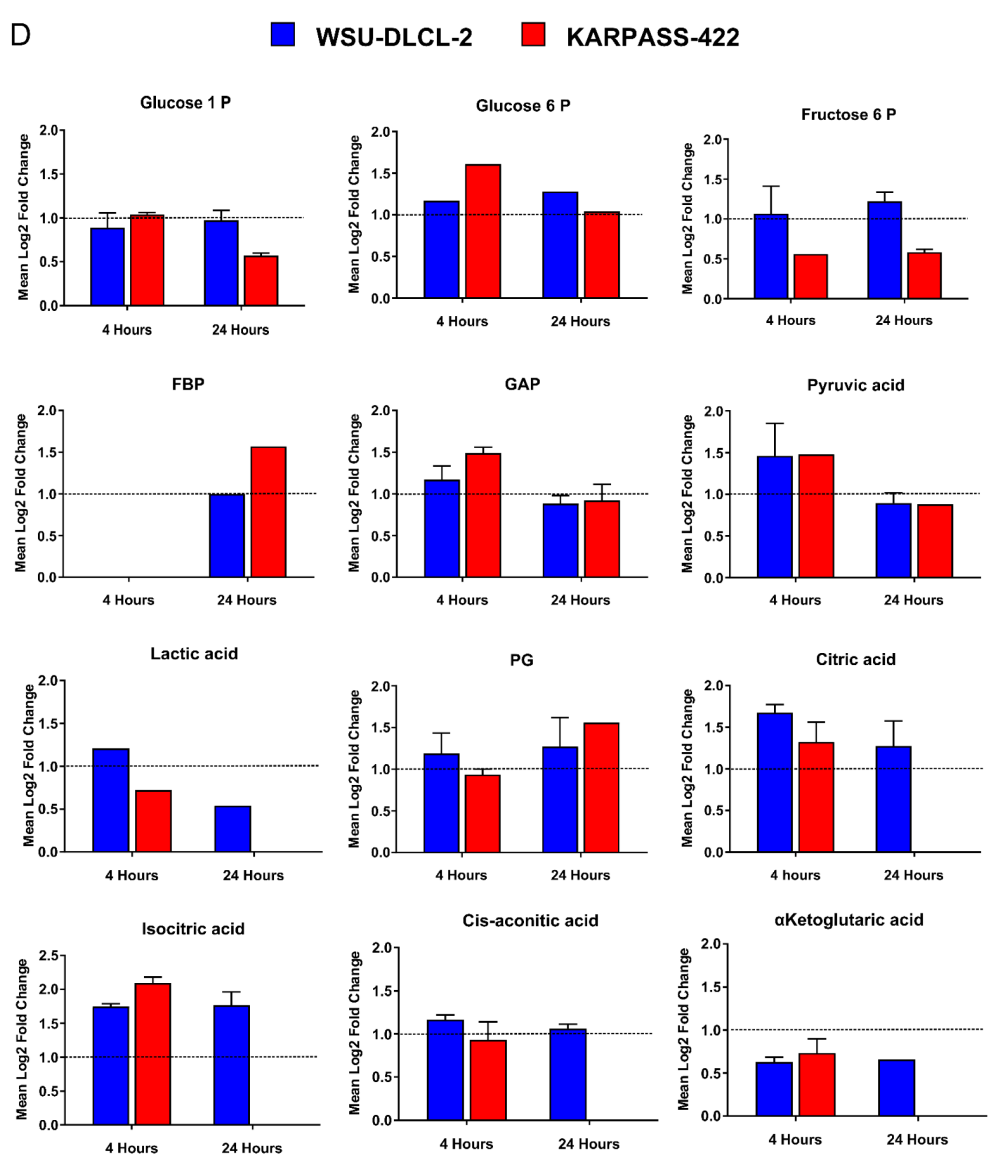
AZD3965 inhibits lactate transport and impacts global metabolic pathways **A.** Raji *in vitro* conditioned media lactate 4 hr post AZD3965. IC_50_ +/- 95% confidence intervals. Data representative of 3 independent experiments. Spent media lactate was measured by LC-MS. **B.** Raji *in vitro* intracellular lactate following 4 hr and 24 hr 100 nM AZD3965. Data representative of 2 independent experiments. Intracellular lactate was measured by LC-MS. **C.** Heat map listing the number of WSU-DLCL-2, Karpas-422 and HT validated metabolites significantly modulated ( [log_2_ Fold change] > 0.5, QC CV<30% and p-value<0.05) per metabolic pathway following 4 hr or 24 hr 100 nM AZD3965. Comparison to the relevant non treated control group. Red colour represents most impacted metabolic pathways while blue represents minimal pathway perturbation. Pathway score describes the number of significantly modulated metabolites per metabolic patway. **D.** Modulation of individual glycolytic and TCA cycle metabolites for the Karpass-422 and WSU-DLCL-2 cell lines 4 hr and 24 hr post 100 nM AZD3965. Y axis = Metabolites significantly modulated ( [log_2_ Fold change] > 0.5, QC CV<30% and p-value<0.05). (Glucose-1-P; glucose-1-phosphate, Glucose-6-P; glucose-6-phosphate, Fructose-6-P; fructose-6-phosphate, FBP; Fructose-1-6-Bis-Phosphate, GAP; glyceraldehyde-3-phosphate, PG; 6-phosphogluconate, aKET; α-ketoglutarate).

In MCT1 positive cell lines with a high dependency on glycolysis, AZD3965 would be predicted to impart its phenotypic efficacy through inhibiting lactate transport, thereby promoting intracellular acidification and metabolic catastrophe. Indeed, AZD3965 inhibited Raji lactate efflux with an IC_50_ of 5.12 nM (95% CI 3.76 - 6.97) as determined through measuring spent media lactate by LC-MS 4 hr post AZD3965 treatment (Figure 4A). Maximum inhibition of Raji lactate efflux was demonstrated by 100 nM AZD3965. As demonstrated by Figure 4B and consistent with inhibition of lactate efflux inhibition, 100 nM AZD3965 significantly increased Raji intracellular lactate 4 hr post treatment, but not at a later 24 hr timepoint.

We investigated whether inhibition of MCT-1 mediated lactate transport by AZD3965 resulted in wider intracellular metabolic deregulation. The impact of MCT1 inhibition on the intracellular metabolic profile of the sensitive cell lines WSU-DLCL-2 and Karpas-422 and the insensitive cell line, HT was measured through LC-MS metabolomic profiling of central carbon metabolites following 4 hr and 24 hr with 100 nM AZD3965 (Figure 4C). Strong deregulation of glycolysis, gluconeogenesis and the pentose phosphate pathway and the citrate cycle was demonstrated in the WSU-DLCL-2 and the Karpas-422 cell lines whilst minimal perturbation was seen for the HT cell line. As demonstrated in Figure 4D*,* 4 hr and 24 hr 100 nM AZD3965 treatment in the Karpass-422 and WSU-DLCL-2 cell lines induced significant up regulation of key glycolytic and gluconeogenic intermediates including gluose-6-phosphate, fructose-6-phosphate, fructose-1-6-phosphate, glyceraldehyde-3-phosphate, pyruvate and lactate. Significant enrichment of TCA cycle metabolites including citrate and iso-citrate was also demonstrated (Figure 4D). Collectively, these data demonstrate that in sensitive cell lines, AZD3965 directly inhibits lactate transport, causing an acute increase in intracellular lactate levels followed by glycolytic feedback and increased flux into the TCA cycle.

### AZD3965 modulates tumor lactate and inhibits *in vivo* growth of the Raji xenograft model

**Figure 5:**
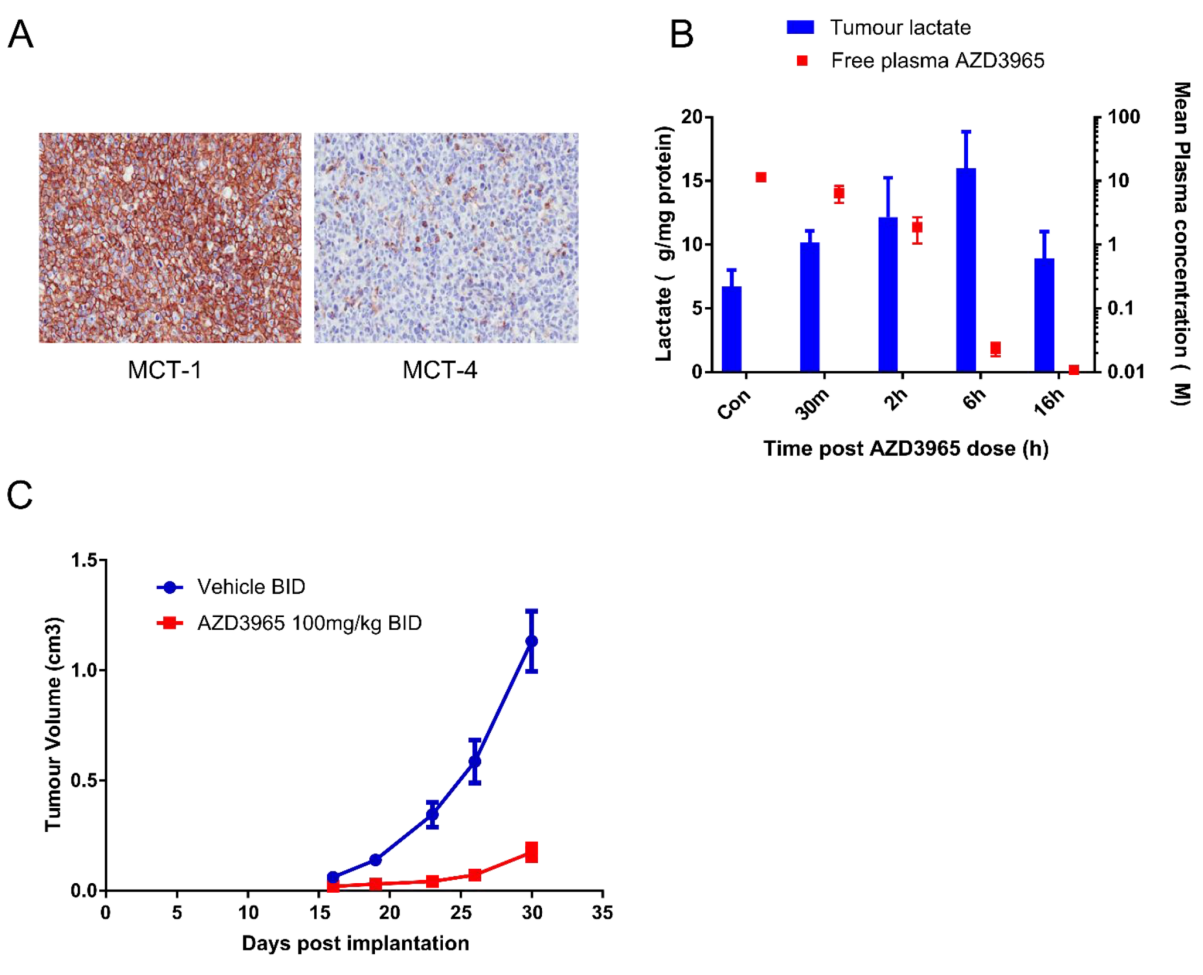
AZD3965 inhibits Raji DLBCL lactate transport and xenograft growth **A.** MCT1 and MCT4 protein expression assessed by immunohistochemisty in Raji tumor sections after 20 days dosing. **B.** The relationship between Raji tumor lactate accumulation and free plasma AZD3965 concentration following a single oral administration of 100 mg/kg AZD3965 at the indicated time points. For each time point 5 tumors were used for the analysis. (Solid bar: tumor lactate, solid square: free plasma AZD3965). **C.** Raji tumor bearing mice were treated orally with 100 mg/kg AZD3965 twice daily (BID) for 15 days before dosing was stopped and recovery of tumor growth was monitored. Geometric mean tumour volume +/- SEM are shown.

The pharmacodynamic activity of AZD3965 was investigated in the Raji xenograft model. To confirm the predominant MCT1 expression *in vivo*, Raji tumor sections were stained with antibodies to MCT1 and MCT4 (Figure 5A). The Raji xenograft model demonstrates strong MCT1 membrane expression on tumor cells whereas MCT4 was not expressed in the Raji tumor cells but was observed in fibrovascular cells. The pharmacodynamic activity of AZD3965 was investigated in the Raji xenograft model. Following a single dose of 100 mg/kg AZD3965 led to accumulation of lactate in Raji tumors within 30 min of compound administration (Figure 5B), consistent with the expected mode of action. A significant increase in tumor lactate was observed at 2 hr, consistent with the high free plasma concentration of AZD3965 at these time points (1200- and 300- fold cover over *in vitro* cell proliferation IC_50_ at 30 min and 2 hr respectively). At 24 hr after a single oral dose, tumor lactate concentrations were similar to that in control tumors consistent with the low free plasma concentration of AZD3965 at 24 hr. To determine AZD3965 monotherapy efficacy, anti-tumour activity was assessed in the Raji xenograft model. Twice daily oral administration of AZD3965 at 100mg/kg led to significant tumor growth inhibition (85%, p<0.0001; Figure 5C) when compared with vehicle treated controls. Collectively, these data confirm that in the high MCT1 expressing Raji xenograft model, MCT1 inhibition leads to tumor cell lactate accumulation and a reduction in Raji tumor growth.

### Combination targeting of MCT1 and GLS1 promotes tumor cell apoptosis *in vitro*

**Figure 6:**
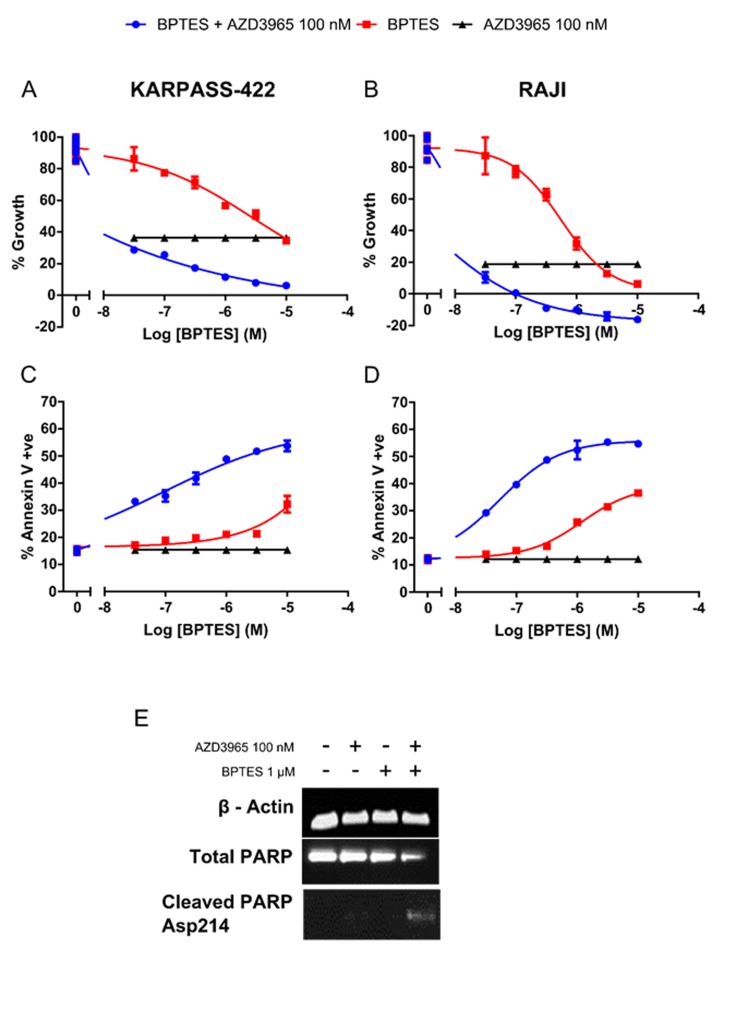
Combination targeting of MCT1 and GLS promotes *in vitro* DLBCL apoptosis **A.** Karpas-422 and **B.** Raji viable cell number 72 hr post concurrent AZD3965 and BPTES combination treatment. **C.** Karpas-422 and **D.** Raji Annexin V staining 72 hr post concurrent AZD3965 and BPTES treatment. (All combination data points were significantly different to both agents alone p<0.05). **E.** Combination targeting of MCT1 and GLS induces Raji apoptosis as assessed through whole cell lysate cleaved PARP 48 hr post 100 nM AZD3965 and 1 μM BPTES. Data representative of 3 independent experiments.

Glutamine represents an alternate carbon source to glucose, with Glutaminase (GLS) converting glutamine to glutamate for subsequent entry into the TCA cycle. The HBL-1, HT, TMD8, WSU-DLCL-2 DLBCL cell lines and Karpas-422 NHL cell lines and the Raji Burkitt’s lymphoma cell line all demonstrate phenotypic sensitivity to the GLS inhibitor BPTES (Supplementary Figure 1). AZD3965 when dosed simultaneously with BPTES co-operatively decreases cell number and induced apoptosis as measured through Annexin V staining. This was seen in cell lines both exquisitely phenotypically sensitive to AZD3965 monotherapy (Raji) or with reduced relative phenotypic sensitivity to AZD3965 (Karpas-422). In the Karpas-422 cell line, the concurrent combination of AZD3965 and BPTES resulted in a significantly greater, although still partial, reduction in cell growth as compared to either AZD3965 or BPTES alone. Annexin V staining was significantly increased in the combination (48.85% +/- s.e. 0.65) as compared to AZD3965 (15.38% +/- s.e. 0.34) or BPTES (21.10% +/- s.e. 0.50) alone (Figure 6A & 6C). In the Raji cell line, the concurrent combination of AZD3965 and BPTES resulted in a complete reduction in cell growth as compared to either agent alone. Annexin V staining was significantly increased in the combination (52.40% (+/- s.e. 2.40) as compared to AZD3965 (12.23% +/- s.e. 0.25) or BPTES (25.75% +/- s.e. 0.05) alone (Figure 6B & 6D). Further induction of cell death was confirmed in the Raji cell line by exposing the cells 48 hr to the concurrent combination of AZD3965 and BPTES and monitoring cleaved PARP (Figure 6E). These data demonstrate that the combination of MCT1 and GLS1 inhibition results in enhanced inhibition of cell growth and increased cell death in lymphoma cell lines.

### AZD3965 in combination with doxorubicin or rituximab results in enhanced inhibition of Raji xenograft growth

**Figure 7:**
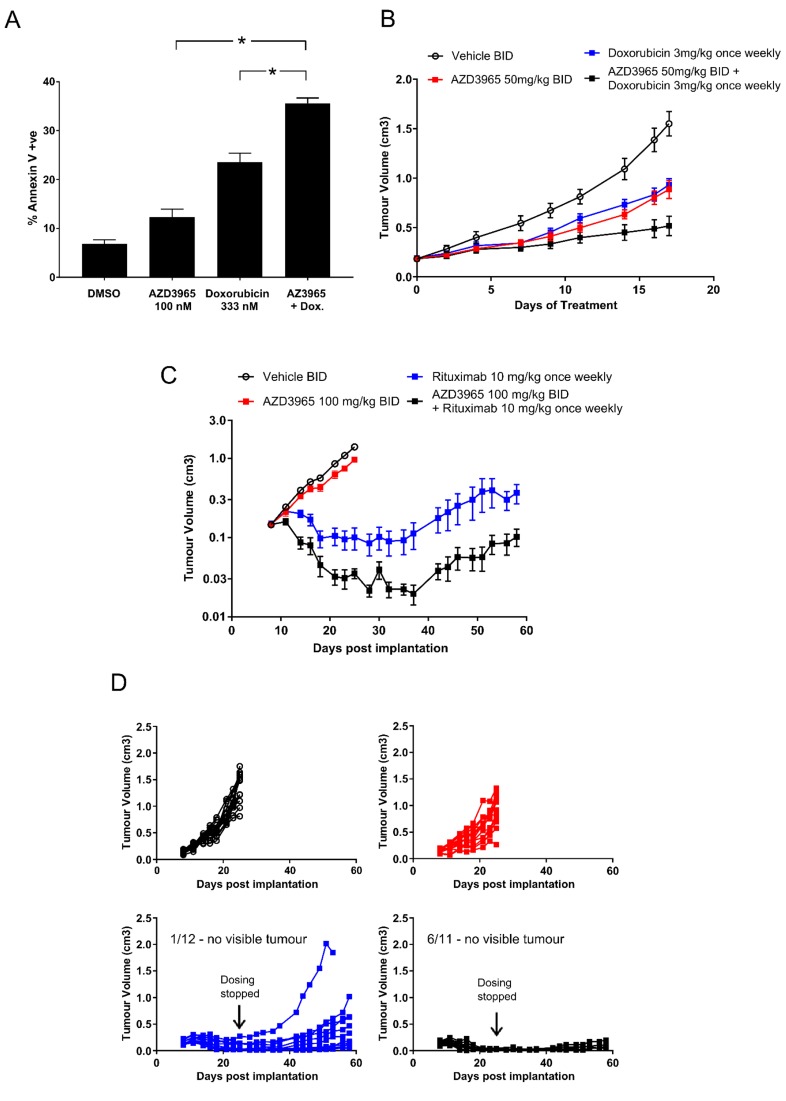
Enhanced AZD3965 Raji xenograft efficacy in combination with doxorubicin or rituximab **A.** The *in vitro* sequential dosing of AZD3965 and doxorubicin increases Annexin V staining in Raji cells (* p<0.05). (Data representative of 2 independent experiments). **B.** Enhanced efficacy observed when combining AZD3965 with doxorubicin in the Raji xenograft model. Raji tumor bearing mice were treated with either vehicle, 50 mg/kg AZD3965 or 3 mg/kg doxorubicin alone or a combination of AZD3965 and doxorubicin as indicated. AZD3965 was administered twice daily (BID) and doxorubicin was administered once weekly. Compared to vehicle only control, the total growth inhibition following AZD3965 alone 52% *(p = 0.0005),* doxorubicin alone 47% *(p = 0.0001*) and the combination of AZD3965 and doxorubicin 81% *(p = <0.0005)*. **C.** Enhanced efficacy observed when combining AZD3965 with rituximab in the Raji xenograft model. Raji tumor bearing mice were treated with either vehicle, 100 mg/kg AZD3965 or 10 mg/kg rituximab alone or a combination of AZD3965 and rituximab as indicated. AZD3965 was administered twice daily and rituximab was administered once weekly. Cessation of dosing in this study is indicated, and where tumours are measurable (Rituximab monotherapy *n* = 11, Rituximab and AZD3965 *n* = 5) the geometric mean tumour volume +/- SEM are shown. Non-measurable tumours prevents standard statistical analysis.

DLBCL and Burkitt’s lymphoma are commonly treated with R-CHOP of which doxorubicin and rituximab are active components. To determine the impact of lactate transport inhibition and/or glycolytic deregulation on phenotypic sensitivity to doxorubicin *in vitro*, Raji cells were pre-incubated 24 hr with 100 nM AZD3965 prior to 4 hr transient 333 nM doxorubicin exposure followed by continuous 100 nM AZD3965. Doxorubicin concentrations were selected based upon observed peak (∼5 μM) and steady state (25-250 nM) plasma concentrations observed in patients after standard bolus doxorubicin infusion (35-75 mg/m^2^) [34, 35]. The number of Annexin V positive cells in the AZD3965 plus doxorubicin schedule (35.57% +/- s.e. 0.65) was significantly greater than in either of the AZD3965 (12.29% +/- s.e. 0.48) or doxorubicin (23.57% +/- s.e. 1.05) monotherapies alone (Figure 7A). We sought to determine the *in vivo* efficacy of combining AZD3965 with either doxorubicin or rituximab in the Raji xenograft model. Since dosing 100 mg/kg AZD3965 twice daily in combination with doxorubicin was not well-tolerated due to body weight loss, we therefore decreased the dose for this combination study to 50 mg/kg twice daily. Inhibition of tumor lactate transport was still observed at this dose level (data not shown). In Raji tumors, the combination of 50 mg/kg twice daily AZD3965 with 3 mg/kg once weekly doxorubicin, significantly enhanced tumor growth inhibition compared with either monotherapy activity alone with the combination group achieving 81% tumor growth inhibition when compared to vehicle treated controls (p<0.0001 Figure 7B).

We then assessed the *in vivo* efficacy of AZD3965 in combination with rituximab in the Raji xenograft model. At the end of the dosing period (17 days) rituximab monotherapy and the combination of AZD3965 with rituximab led to tumour regressions, with 6 of 11 tumors showing complete responses in the combination group compared to 1 of 12 tumors in the Rituximab monotherapy group (Figure 7C). To explore the duration of the response the monotherapy and combination groups remained on study after cessation of dosing for a further 33 days. During this period the complete responses observed in both the monotherapy and combination groups were maintained however, within the Rituximab monotherapy group tumors began to regrow. (Figure 7C). It should be noted that monotherapy efficacy achieved with AZD3965 when dosing established tumors was moderate compared with commencing dosing immediately post-tumor cell implantation. AZD3965 in combination with either doxorubicin or rituximab was well tolerated. These data demonstrate that in Raji Burkitt’s lymphoma tumors AZD3965 can be combined effectively with doxorubicin or rituximab.

## DISCUSSION

AZD3965 is a potent inhibitor of MCT1 with 6-fold selectivity *versus* MCT2 and with excellent selectivity *versus* MCT3 and MCT4. AZD3965 inhibitors demonstrate potent monotherapy activity across a number of DLBCL, NHL and Burkitt’s lymphoma cell lines. AZD3965 EC_50_ <100 nM was identified in a number of cell lines with near complete loss of cell number. DLBCL, Burkitt’s and other lymphoma often display MYC rearrangements and to a lesser extent gene amplifications [36]. It is well established that MYC contributes to the metabolic reprogramming of tumors [37] and that MYC regulates the expression of a number of glycolytic genes including *SLC16A1* (gene encoding MCT1) [16]. The high sensitivity of lymphoma cell lines could be due, in least in part, to the high frequency of MYC translocation/amplification. Other studies have evaluated the activity of MCT1 inhibitors in SCLC, an alternate tumor setting where frequent MYC amplifications are observed. AZD3965 decreased the proliferation of SCLC cell lines and inhibited the growth of the COR-L103 xenograft model *in vivo* [38].

A number of studies have identified MCT4 lactate transport as a potential resistance mechanism to MCT1 inhibition [16, 38, 39] suggesting that where MCT4 is present, the tumor may be refractory to AZD3965. Indeed, across the cell lines tested here *in vitro*, AZD3965 was most active in cell lines displaying a MCT1 positive and MCT4 negative profile. The two cell lines relatively resistant to AZD3965 monotherapy activity, HBL-1 and HT were shown to express both MCT1 and MCT4 suggesting that MCT4 may be continuing to drive lactate transport even in the presence of AZD3965.

Consistent with AZD3965 inhibition of MCT1 lactate transport, intracellular lactate was increased and extracellular level decreased following AZD3965 treatment of the Raji cell line in *in vitro* culture. We have additionally demonstrated in two AZD3965 phenotypically sensitive cell lines, WSU-DLCL-2 and Karpas-422, that a number of intracellular glycolytic intermediates are raised along with strong deregulation of the central carbon metabolism (glycolysis, gluconeogenesis and the pentose phosphate pathway and the TCA cycle) following AZD3965 treatment, but not in the AZD3965 phenotypically resistant HT MCT1 and MCT4 positive cell line. These data suggest that in sensitive cell lines, MCT1 inhibition results in feedback inhibition of glycolysis accompanied by an increase in flux to the TCA cycle.

Treatment of Raji xenografts with AZD3965 induced an increase in lactate in a time-dependant manner which correlated with free plasma exposure consistent with inhibition of lactate efflux out of the tumor cell. In addition, AZD3965 led to significant efficacy as a single agent in the Raji xenograft model. Doherty *et al.* have also demonstrated that the inhibition of MCT1 decreased the proliferation of the Raji Burkitt lymphoma cell line *in vitro* and delayed the onset and penetrance of Raji tumor growth *in vivo* [16]. The degree of tumor growth inhibition was different depending on the timing of treatment. When dosing commenced a few days post tumor cell implantation this resulted in tumor stasis. However, the same treatment on established tumors led to moderate efficacy. One potential hypothesis to explain this observation is that the metabolic phenotype of tumors, during the initiation of growth, are metabolically constrained rendering these tumors more susceptible to metabolic pathway intervention. Further studies are required to fully elucidate the impact of tumor size on efficacy.

R-CHOP based treatment regimens are currently standard-of-care in DLBCL and Burkitt’s lymphoma. We have demonstrated here that pre-incubation *in vitro* of the Raji Burkitt’s lymphoma cell line with AZD3965 sensitises the cells to subsequent doxorubicin exposure as measured by increased Annexin V staining and PARP cleavage. Doxorubicin, an anthracycline antibiotic, promotes DNA strand breakage so thereby preventing DNA replication through DNA intercalation and covalent DNA binding and through also acting as a topoisomerase II poison [34, 40]. Here, Raji cells *in vitro* were dosed over a 4 hr period with doxorubicin 550 nM. Similar doxorubicin concentrations have also been linked to the inhibition of DNA synthesis *in vitro* in HeLa cells [35] and the MCF-7 breast tumor cell line [41]. Doxorubicin has been shown to increase poly(ADP-ribose) polymerase (PARP) activity in the MCF-7 and MDA-MB231 cell lines in a time- and dose- dependent manner [42, 43]. Upon persistent PARP activation, the activated enzyme is capable of depleting cellular pools of NAD+ an energetically demanding process. One hypothesis may be that AZD3965 sensitises cells to doxorubicin through glycolytic shut-down and thereby reducing the capacity of the cell to maintain cellular NAD+ and ATP levels upon subsequent exposure to doxorubicin. Furthermore, significantly enhanced efficacy has also been observed when combining AZD3965 with doxorubicin in the Raji xenograft model. AZD3965 dosed concurrently with doxorubicin led to improved Raji tumor growth inhibition than either single agent alone. In contrast to the *in vitro* combination data where significant cell death was observed, the *in vivo* efficacy did not cause tumor stasis. Interestingly, recent studies have demonstrated that lonidamine, a cooperative inhibitor of MCT1, 2 and 4 also potentiates the efficacy of doxorubicin *in vitro* and *in vivo* [44]. Moreover, Nath *et al.* demonstrate that lonidamine causes intracellular acidification and propose that potentiation of doxorubicin could result from cation trapping of the weakly basic anthracycline [44]. Further studies to fully understand the mechanism of action of the combination, and to determine whether scheduling *versus* concurrent dosing delivers greater tumor growth inhibition, are required.

We have demonstrated here that AZD3965 can be combined with rituximab in the Raji xenograft model and that this combination was well tolerated, resulting in significant anti-tumor efficacy compared to rituximab alone. Indeed, in the combination group the tumors were either non- measurable or growth was static whereas in the rituximab monotherapy group almost all tumors showed signs of re-growth following withdrawal of treatment. It is likely that agents targeting MCT1 will give greatest benefit in combination with the R-CHOP regimen in DLBCL. On-going trials in DLBCL include Bortezomib (targeting NF-kB), Ibrutinib (targeting BTK) and Lenalidomide (an immunomodulatory agent) in combination with R-CHOP and as maintenance therapies [45].

Inhibition of lactate transport and reduction in glycolytic flux may render cells more sensitive to other key metabolic pathways such as glutaminolysis to feed the TCA cycle. Indeed, glucose deprivation of the P494 human Burkitt’s lymphoma model demonstrated that glutamine-derived fumarate, malate and citrate were significantly increased [46] consistent with increased glutaminolytic flux. Furthermore, other studies have demonstrated that haematological cell lines also utilize glutamine as a key carbon source. Across a study of 41 multiple myeloma, acute lymphocytic leukemia and chronic lymphocytic leukemia cell lines, glutamine deprivation resulted in a minimum 70% reduction in growth, inducing cell death in 11 of the cell lines [47]. CB-839 (glutaminase inhibitor, inhibiting recombinant GLS with an IC_50_ ∼30 nM) exhibited an anti-proliferative IC_50_ <100 nM *in vitro* across 36 of the cell lines, whilst twice daily CB-839 dosing also resulted in a 71% decrease of RPMI-8226 (multiple myeloma) tumor volume. The observation that enhanced apoptosis is observed in Raji and Karpass-422 cells treated with a concurrent dosing of AZD3965 and BPTES suggests that the combined inhibition of the glycolytic/lactate transport and glutaminoloysis pathway promotes metabolic catastrophe and cell death. Further studies are required to explore the tolerability and efficacy of combining MCT1 with GLS1 inhibitors in pre-clinical xenograft models.

Together, these data support the clinical evaluation of AZD3965 in MCT1 expressing DLBCL, NHL and Burkitt’s lymphoma either as monotherapy or in combination with either metabolic pathway inhibition and/or incorporation into R-CHOP based strategies. Our findings also support the targeting of multiple DLBCL metabolic pathways and further investigation into future combination strategies of AZD3965 with targeted inhibitors of GLS1. We also propose that selecting patients based upon a MCT1 positive/MCT4 negative expression profile may be predictive to AZD3965 clinical activity. AZD3965 is currently being tested in a phase 1 clinical trial.

## MATERIALS AND METHODS

### AZD3965 MCT1 binding affinity

The binding affinity (pKi/Ki) of AZD3965 to Jurkat cell membranes (which express MCT1) was measured using a scintillation proximity assay (SPA) as described previously [30]. Wheatgerm agglutinin SPA beads were coated with Jurkat cell membranes and incubated with [^125^I]-MCT1 ligand and the binding affinity of test compounds determined in competition binding studies.

### AZD3965 MCT2 selectivity assays

The selectivity of AZD3965 was determined in filter binding assays measuring the Ki to yeast membranes expressing recombinant human MCT1 or human MCT2 by displacement of a [^3^H]-MCT1 or MCT2 ligand [30].

### AZD3965 inhibition of lactate membrane transport

To determine the selectivity of AZD3965, the effect on lactate transport mediated by MCT3 or MCT4 was determined. Human MCT1, MCT3 or MCT4 were expressed in the MCT1, MCT2, MCT3 and MCT4 null rat pancreatic INS1 cell line. Lactate transport was measured using the pH- sensitive fluorescent dye BCECF ((2’,7’-Bis-(2-Carboxyethyl)-5-(and-6)-Carboxyfluorescein, Acetoxymethyl Ester)) to detect the rapid decrease in intracellular pH that accompanies proton-linked lactate transport as described by Murray *et al.* [30]. Briefly, cells were loaded with 1 mM BCECF and incubated with compound or vehicle for 1 hr in Tyrodes buffer pH7.4. The ratio of fluorescence at 535 nm after excitation at 490 nm/440 nm was measured after addition of exogenous L-lactate using the Flexstation scanning fluorometer. The change in pHi was determined from a calibration curve using buffer containing nigericin to equilibrate internal and external pH [30].

### Cell culture

HBL-1, HT, TMD8 and WSU-DLCL-2 DLBCL cell lines, SU-DHL-10 and Karpas-422 NHL cell lines and the Raji Burkitt’s lymphoma cell line were obtained from the American Type Culture Collection. Cells were cultured and unless otherwise specified assayed in RPMI 1640 (Sigma) supplemented with 10% v/v fetal bovine serum and 2 mM L-glutamine at 37°C in a humidified atmosphere of 5% CO_2_.

### Compound dosing

For monotherapy dosing, cells were exposed to concentrations of AZD3965 or BPTES [bis-2-(5-phenylacetamido-1,2,4-thiadiazol-2-yl)ethyl sulfide), a potent inhibitor of the kidney GLS (KGA) isoform, but not of the liver GLS (GCA) isoform [48] ranging from 10 - 0.003 μM or DMSO vehicle control. For the concurrent combination of AZD3965 and BPTES, the drugs or DMSO vehicle control were added simultaneously at their respective concentrations. For the AZD3965 and doxorubicin (Sigma) scheduling, cells were pre-treated with 100 nM AZD3965 or DMSO vehicle control for 24 hr. Cells were subsequently dosed with doxorubicin for 4 hr. The cells were then washed twice with PBS, re-suspended in fresh media and re-dosed with 100 nM AZD3965 or DMSO vehicle control for the duration of the assay.

### Western blot analysis

For basal untreated cell lysates, cells were seeded into T25 flasks at 0.5x10^6^ cells/ml for 24 hr before harvesting. For treatment with AZD3965 or BPTES monotherapy or the concurrent combination of AZD3965 and BPTES cells were seeded into 12-well plates at 0.5x10^6^ cells/ml and compound dosed immediately. Cell lysates were prepared through lysing cell pellets on ice with a buffer containing 25 mM Tris-HCl, 3 mM EDTA, 3 mM EGTA, 50 mM NaF, 2 mM sodium orthovanadate, 0.27 mM Sucrose, 10 mM β-glycerophosphate, 5 mM sodium pyrophosphate and 0.5% (v/v) Triton X-100 and protease and phosphatase inhibitors (Sigma). Cell lysates to be probed for PARP, cleaved PARP and β-acting loading control were then diluted with sample loading buffer (Invitrogen). Cell lysates to be probed for MCT1 and MCT4 were further treated for 60 min on ice with equal volumes of buffer containing 150 mM NaCl, 100 mM EDTA, 50 mM HEPES pH 7.5 and 2% (w/v) CHAPS detergent. The cell lysates were then diluted with sample loading buffer containing 125 mM Tris-HCl pH 6.8, 4% (w/v) SDS, 20% (v/v) glycerol, 50 mM Dithiothreitol and β-mercaptoethanol. Lysates were separated on 4 to 12% Bis-Tris Novex gels and transferred onto nitrocellulose membranes. Membranes were blocked with 5% (w/v) non-fat milk in phosphate buffered saline + Tween 20 (3.2 mM Na_2_HPO_4_, 0.5 mM KH_2_PO_4_, 1.3 mM KCl, 135 mM NaCl, 0.05% Tween 20, pH 7.4) and probed overnight with antibodies for PARP (Cell Signaling Technology, Inc.), cleaved PARP (BD Biosciences), MCT1 (in-house generated polyclonal), MCT4 (in-house generated polyclonal) and β-actin loading control (Sigma). Membranes were then washed and incubated with horseradish peroxidase-tagged secondary antibodies, followed by detection on a Syngene ChemiGenius with Super Signal West Dura Chemiluminescence Substrate.

### Cell proliferation

Cell proliferation was determined by 2 methods, CellTiter 96 Aqueous One Solution Cell Proliferation Assay (MTS, Promega) and Flow Cytometry. Cells were seeded in 96-well plates at a density to allow for logarithmic growth during the 72 hr assay and compound dosed accordingly. For the MTS endpoint, cell proliferation was measured by the CellTiter Aqueous One Solution reagent in accordance with the manufacturer’s protocol. For Flow Cytometry, cell number was measured by Guava Viacount Reagent for Flow Cytometry (Merck Millipore) on the GUAVA easyCyte Flow Cytometer in accordance with the manufacturer’s protocol. Pre-dose measurements were made. The concentrations of drug required to reduce the growth of treated cells to half that of untreated cells (GI_50_) were determined.

### Annexin V staining

Cells were seeded in 96-well plates at a density to allow for logarithmic growth during the 72 hr assay and compound dosed accordingly. Annexin V positive cells were determined using the Guava Nexin Reagent for Flow Cytometry (Merck Millipore) on the GUAVA easyCyte Flow Cytometer in accordance with the manufacturer’s protocol. The cell impermeant dye, 7-ADD is used as an indicator of cell membrane structural integrity. Annexin V-PE was used to detect phosphatidylserine on the external membrane of apoptotic cells. The % Annexin V positive cells were calculated.

### Metabolite extraction of *in vitro* intracellular material and conditioned culture medium

Cells were re-suspended into RPMI 1640 (Sigma) supplemented with 10% v/v stem cell screened, dialysed fetal bovine serum for SILAC (Thermo Scientific) and 2 mM L-glutamine and seeded into 24-well plates at 0.75x10^6^ cells/ml. Cells were immediately exposed to AZD3965 or DMSO vehicle control. At the appropriate time points the contents of each well were transferred to an eppendorf tube and centrifuged at 13,000 rpm for 2 min. 100 μl media supernatant was aliquoted into an eppendorf tube and proteins were immediately precipitated with 400 μl ice cold 50:50 acetonitrile:methanol for 5 min. Following centrifugation at 13,000 rpm for 5 min the clarified supernatant was then transferred to an eppendorf tube and stored at -20°C until analysis. The remaining media supernatant was discarded and the cell pellet immediately quenched with 400 μl ice cold 40:40:20 acetonitrile:methanol:water for 5 min. The eppendorf tubes were then centrifuged at 13,000 rpm for 5 min to pellet precipitated proteins and cell debris. Clear supernatants were transferred to clear eppendorf tubes and stored at -20°C until analysis.

### Liquid chromatography tandem mass spectrometry

Intracellular and conditioned media metabolites were analysed following chromatographic separations and tandem mass spectrometric detections consisting of an Ultimate 3000RS chromatographic system (Thermo, UK) hyphenated to an AB4000 triple quantropole (ABSCIEX, UK) mass spectrometer operating on negative ion mode. Metabolites were resolved following a gradient elution profile on an UPLC HSS T3 C18 1.8 μM, 2.1 x 1000 mM column under temperature (60°C) controlled conditions. The binary solvent system consisted of buffer A (H_2_0, 10 mM tributylammonium, 15 mM acetic acid) and buffer B (MeOH/Isopropanol 80/20) operated in a flow rate of 400 μL/min at a time schedule of: 0 min, 0% B; 0.5 min, 0% B; 4 min, 5% B; 6 min, 5% B; 6.5 min, 20% B; 8.5 min, 20% B; 14 min, 55% B; 15 min, 100% B; 18 min, 100% B; 18.01 min, 0% B; 21 min 0% B. Further details regarding sample preparation and metabolite analysis by liquid chromatography coupled to tandem mass spectrometry can be found as detailed by Michopoulos et al [49]. For lactate quantitation the above solvent system was adjusted to isocratic elution of 20% B (0-3 min) followed by 2 min column washing with 100% B before the column was re-equilibrated to initial conditions resulting in an overall sample analysis time of 8 min. For lactate analysis cell extracts and media samples were diluted with ultrapure water in a ratio of 1/10 and 1/100 (extract:final volume) respectively. ^13^C lactate spiked at a concentration of 5 μM was used as an internal standard to normalise for analytical variability. Lactate quantitation was obtained for the linear range 0.5 to 160 μM on both matrices. Collected data were subject to media value normalisation and modulated metabolites between treated and control group were flagged using unit variate statistical analysis (ttest, p value <0.05). Further validation on the significance of the metabolic modulation was obtained using the co-efficient of variation of the analytical measurement (QC CV <30%) and the magnitude of the metabolite level change across treated and control groups ( [Log_2_(average peak area treated / average peak at control]) > 0.5). Significantly modulated metabolites were annotated to KEGG metabolic pathways using an in-house interface to enable pathway visualisation of the determined metabolic perturbation. The overall number of significantly modulated metabolites (pathway score) was used to summarise the metabolic perturbation seen in each of the cell lines.

### Immunohistochemistry and immuno-cytochemistry

For all IHC and staining, tissues were sectioned at 4 μm, mounted on positive charged glass slides and dried at 37°C overnight. Sections were dewaxed in xylene and rehydrated in graded alcohols and water. All incubations were performed at room temperature and washes performed with TBS containing 0.05% Tween (TBST). Heat-mediated antigen retrieval was performed in a RHS-2 microwave vacuum processor (Milestone) at 110°C for 5 min, in pH 8 EDTA retrieval buffer. Sections were cooled in running tap water for 20 min. For all antibodies, endogenous peroxidase activity was blocked with 3% hydrogen peroxide for 10 min and then non-specific binding sites were blocked with serum-free protein block (X0909, Dako) for 20 min. Sections were incubated for 1 hr with either MCT1 polyclonal antibody (generated in-house, 1:3000) or MCT4 polyclonal antibody (generated in-house, 1:3000). A polymer detection system (Dako Envision +K4007) was used for secondary detection and following washing in TBST, sections were incubated in diaminobenzidine for 10 min (K3466, Dako) and counterstained with Carazzi’s haematoxylin.

### *In vivo* Raji anti-tumor studies

All studies involving animals were performed in accordance with institutional guidelines in AALAAC accredited labs in the United States as well as in the United Kingdom in full accordance with the UK Home Office legislation, Animal Scientific Procedures Act 1986. All mice used were female SCID mice between 8 to 12 weeks old. Raji tumor cells (5x10^6^ cells in PBS) were implanted subcutaneously in a total volume of 0.1 ml/mouse. For the monotherapy study, animals were randomised into vehicle or treatment groups 7 days after cell implantation and dosed orally for 15 days. Dosing was stopped after 15 days and thereafter animals remained on study for a further 10 days to assess re-growth. For the combination studies animals were randomised into vehicle and treatment groups when mean tumor volume reached approximately 0.2 cm^3^. For all studies the treatment groups received AZD3965 at either 50mg/kg or 100mg/kg orally twice daily as monotherapy or in combination with 3mg/kg Doxorubicin (Sigma) dosed intravenously or 10mg/kg Rituximab dosed intraperitoneally. AZD3965 was prepared in 0.5% Hydroxy propyl methyl cellulose/0.1% polysorbate-80. Tumor volume was measured bilateraly by caliper, animal body weight, and tumor condition were recorded twice weekly for the duration of the study. Tumor volume was calculated using the formula π*/6000 x length x width^2^. Growth inhibition from the start of treatment was assessed by comparison of the geometric mean change in tumor volume for the control and treated groups. Statistical significance was evaluated using a one-tailed, *t* test.

### Assessment of tumor lactate levels

When mean tumor volume reached 0.5 cm^3^ Raji tumor-bearing mice were administered a single oral dose of 100 mg/kg AZD3965 or vehicle. Tumor and blood was collected at various time points after dosing. Plasma was prepared from total blood and frozen at -20^o^C. Frozen tumor samples were lysed in 1x cell lysis buffer (Cell Signaling Technology, Inc.) containing phosphatase and protease inhibitors (Sigma) with a Fast Prep Homogeniser (MP Biomedicals). Tumor lysates were cleared by centrifugation. Mouse plasma and tumor lactate was measured using the Trinity Biotech lactate reagent for the quantitative enzymatic determination of lactate. A lactate standard curve allowed for the quantitation of plasma and tumor lactate. Tumor protein was quantitated using a Bio-Rad protein assay. Tumor lactate was then normalised for tumor protein.

### Plasma pharmacokinetic analysis

A stock (2 mM) of the analytical standard was prepared using DMSO and then used on the TECAN to produce spiking solutions. 40 μl of the required blank matrix was then aliquoted into a 96-well plate. The matrix was then spiked with 10 μl of each dilution to give a final concentration range of 1 nM - 10,000 nM. 50 μl of each sample and standards were then quenched with Acetonitrile, mixed and spun in a centrifuge at 3000 rpm for 15 min. 50 μl of the supernatant was then diluted 10-fold with deionised water and the samples analysed using Masslynx on LCMS-MS and processed using Quanlynx.

## SUPPLEMENTARY MATERIALS FIGURE AND TABLE


