# Supplementary material for: Pre-clinical pharmacology of AZD3965, a selective inhibitor of MCT1: DLBCL, NHL and Burkitt’s lymphoma anti-tumor activity
Source: Oncotarget. 2017 May 25;8(41):69219–36. doi: 10.18632/oncotarget.18215 (PMC5642474; doi:10.18632/oncotarget.18215)
Supplement: Supplementary file 1 [file oncotarget-08-69219-s001.pdf]

Pre-clinical pharmacology of AZD3965, a selective inhibitor of MCT1: DLBCL, NHL and Burkitt’s lymphoma anti-tumor activity

Supplementary Material

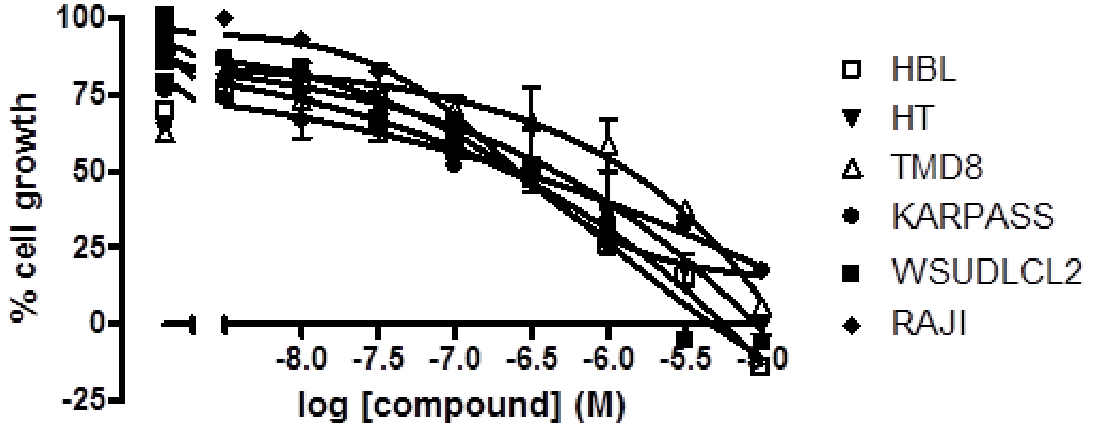

Supplementary Figure 1: BPTES inhibits DLBCL, NHL and Burkitt's lymphoma cell growth as assessed through cell number. Cell number was assessed through cell number cell 72 hr post BPTES treatment. Data representative of 3 independent experiments.

Supplementary Table 1: MYC status of the DLBLC, NHL and Burkitt’s lymphoma cell line panel. (ABC: activated B-cell. GBC: germinal center B-cell).

Cell Line	Type	sIg	MYC status
HBL-1	ABC	IgM	
HT	GCB	IgM	
KARPAS-422	GCB	IgMG	
TMD8	ABC	IgM	
WSU-DLCL-2	GCB	IgG	
Raji			translocation
Su-DHL-10	GCB	IgG	translocation (8;14) (q24;q32)
